# Outcomes of Stevens–Johnson syndrome and toxic epidermal necrolysis in HIV-infected patients when using systemic steroids and/or intravenous immunoglobulins in Pietermaritzburg, South Africa

**DOI:** 10.4102/sajhivmed.v20i1.944

**Published:** 2019-07-04

**Authors:** Antoinette V. Chateau, Ncoza C. Dlova, Halima Dawood, Colleen Aldous

**Affiliations:** 1 Department of Dermatology, School of Clinical Medicine Greys Hospital, University of Kwa-Zulu Natal, KwaZulu-Natal, South Africa; 2 Department Medicine, Infectious Disease Unit, Greys Hospital and Caprisa, University of Kwa-Zulu Natal, KwaZulu-Natal, South Africa; 3 Department of General Medicine, School of Clinical Medicine, University of KwaZulu-Natal, KwaZulu-Natal, South Africa

**Keywords:** Stevens–Johnson syndrome, Toxic epidermal necrolysis, Systemic steroids, Intravenous immunoglobulins

## Abstract

**Background:**

Stevens–Johnson syndrome (SJS) and toxic epidermal necrolysis (TEN) are severe life-threatening mucocutaneous reactions. There is an ongoing controversy regarding the use of systemic corticosteroids and intravenous immunoglobulin (IVIG) in SJS/TEN and their utility in HIV-infected patients.

**Objectives:**

The objective was to assess the outcome of a combination of intensive supportive care with oral corticosteroids in SJS and a combination of systemic steroids and IVIG for 3 consecutive days in HIV-infected patients with TEN. In addition, we assessed management in a general dermatology ward without implementing wound debridement.

**Methods:**

This was a retrospective cohort study of 36 HIV-infected adults with SJS/TEN admitted to a tertiary dermatology unit between 1st January 2010 and 31st July 2011. Standard-of-care protocols included identification and elimination of the possible causative drug, meticulous wound care without debridement, initiation of oral prednisone (1 mg/kg/day) on admission for 3 consecutive days, and the addition of IVIG (1 g/kg/day) for 3 consecutive days to those with TEN.

**Results:**

Of the 36 patients in the study, 32 were female. Nevirapine was the commonest drug implicated. A diagnosis of tuberculosis did not increase the case fatality rate. Complications included infections, anaemia, drug-induced hepatitis, ocular involvement, renal impairment, deep vein thrombosis, respiratory distress, Leucopenia, gastritis and hypernatremia. The overall survival rate was 97%.

**Conclusion:**

HIV-infected SJS and TEN patients were treated in a tertiary dermatology ward with a treatment plan of skin care, and a combination of systemic corticosteroids and IVIG respectively had a survival rate of 97%.

## Introduction

Stevens–Johnson syndrome (SJS) and toxic epidermal necrolysis (TEN) are severe life-threatening mucocutaneous reactions characterised by epithelial sloughing and systemic symptoms.^1,2^ Stevens–Johnson syndrome is characterised by mucous membrane erosions and epidermal detachment, which involves less than 10% of the body surface area (BSA) in the Bastuji-Garin classification. Stevens–Johnson syndrome–toxic epidermal necrolysis overlap represents 10% – 30% BSA involvement, and TEN involves over 30% BSA involvement.^3^ The worldwide incidence rate of SJS is 1.2–6 per million persons per year with a mortality rate of 5%, while the incidence rate of TEN is 0.5–1.2 per million per year with a mortality rate of up to 30%.^4^

In South Africa, there are no published data on the incidence of SJS and TEN. With the current HIV epidemic and increased use of HIV treatment in South Africa, the number of patients with SJS/TEN has increased.^5^ The incidence of SJS/TEN is 1000-fold higher in patients with HIV.^6^ The high incidence of SJS/TEN in immunocompromised patients is likely multifactorial. It may be due to polypharmacy in the management of HIV, slow acetylation of drugs, glutathione deficiency, altered lymphocyte function and cytotoxic sulfamethoxazole metabolites in the case of trimethoprim/sulfamethoxazole.^7,8^ In addition, the high rates of tuberculosis co-infection in individuals with HIV leads to the use of a vast array of drugs to treat these infections. This results in greater susceptibility to SJS/TEN and an associated increase in mortality.^9^

Other factors that contribute to mortality included lymphopenia, neutropenia and hypernatremia, as well as low-serum haemoglobin and hypoalbuminemia.^9,10^

The use of systemic corticosteroids in the treatment of SJS/TEN is controversial.^11^ Systemic steroids in the setting of SJS/TEN has immune-modulating anti-apoptotic effects which downregulate Fas-Fas L binding.^12^ This results in anti-inflammatory properties which inhibit interleukin 2, tumour necrosis factor (TNF) α and interferon (IFN)γ, and immunosuppressant properties which inhibit T cells.^12,13^ Intravenous immunoglobulins (IVIGs) contain anti-Fas antibodies that block the Fas-Fas L interactions on the keratinocyte and thus prevent apoptosis that results in epidermal detachment.^14^ Studies have shown that IVIG arrests disease progression and reduces time to skin healing.^14,15,16^ By combining systemic corticosteroids and IVIG, the inflammatory cascade and the undesirable adverse effects are prevented. Systemic corticosteroids and IVIG abort the inflammatory cascade in SJS/TEN and, hence, the deleterious effects that ensue. Therefore, this retrospective cohort study assessed the outcomes of intensive supportive care combined with systemic corticosteroids and IVIG for 3 consecutive days in HIV-infected patients with TEN. In addition, we assessed the outcome of managing these patients in a general dermatology ward without implementing wound debridement. Some centres treat SJS/TEN as a partial thickness burn^17^ as the clinical presentation is similar to a burn wound, although it is an immune-mediated hypersensitivity reaction. However, SJS/TEN should not be managed strictly as a burn but rather in a specialised dermatology ward without debridement.^1,18,19^ We believe the treatment of SJS/TEN should differ from that of burn treatment because of the different aetiology and pathophysiological mechanism.^1,20^

## Methods

The study was undertaken at the Greys Hospital Department of Dermatology, a tertiary referral centre in Pietermaritzburg, KwaZulu-Natal, South Africa. It is a 530-bed tertiary hospital, serving 3.5 million people in the western part of KwaZulu-Natal.

### Study population

The clinical records of all 39 participants with SJS/TEN admitted to a general dermatology ward from 01 January 2010 until July 2011 were retrospectively reviewed. Three patients were HIV-negative and were thus excluded from the study. All participants were of black African descent.

Participants who presented with organ failure and fulfilled intensive care admission criteria were referred to the intensive care unit where they were co-managed with critical care specialists and the dermatology team.

### Data collection and classification

The review of patient’s clinical records during admission, weekly after discharge for the first month and then monthly for the following 3 months were recorded. Demographics, SCORTEN score, drug history, CD4 count, comorbidities and complications were documented from the records.

SCORTEN (SCORe of toxic epidermal necrosis) is a score used to assess severity and predict mortality in patients with SJS/TEN. It uses seven criteria (Table 1). One point is given to each criterion, and this correlates with a predicted mortality rate.^10^

**TABLE 1:** SCORTEN score.

SCORTEN score	Mortality rate (%)	Criteria
1	3.2	Age > 40 years
2	12.1	Heart rate > 120 bpm
3	35.8	BSA > 10%
4	58.3	Serum urea > 10 mmol/L
≥ 5	90.0	Serum bicarbonate < 20 mmol/L
-	-	Serum glucose > 14 mmol/L
-	-	Cancer or haematological malignancies

*Source*: Bastuji-Garin et al.^10^

BSA, body surface area; bpm, beats per minute.

Criteria used to determine drug causality were timing of the skin lesions after the administration of the drugs (temporality), increase of drug dose, previous history of drug reactions and if the drug reaction occurred when the drug was restarted, criteria noted in the Naranjo scale.^21^ Score of > 9 = definite ADR (adverse drug reaction), 5–8 = probable ADR, 1–4 = possible ADR, 0 = doubtful ADR.

### Treatment plan

The standard-of-care protocols included identifying and eliminating the possible causative drug, initiating oral prednisone (1 mg/kg/day) on admission for 3 consecutive days, and adding IVIG (1 g/kg) for 3 consecutive days to participants with TEN (*n* = 12).

Biochemical assessments on admission included a full blood count, glucose level, and renal and liver function tests. Vital signs were registered every 4 h, and plasma glucose was monitored every 12 h. A screen for sepsis was done when clinically indicated. Fluid depletion and electrolyte abnormalities were corrected, nutritional support was guided by the dietician and pain management was optimised. Oral mucosal care included glycothymol irrigation every 6 h, removal of haemorrhagic crusting and the application of a mixture of prednisolone, remicaine, nystatin and sucralfate (8:8:8:1 formulation) to the lips and oral mucosa. Genital mucosa was treated with daily potassium permanganate sitz baths and lubricated with petroleum jelly (Vaseline ^®^) to prevent adhesions. Pain was controlled with Tramadol hydrochloride 50 mg – 100 mg 6 hourly and paracetamol 1 g 6 hourly. Pethidine was used to alleviate the pain while dressing the wounds. Cutaneous lesions were managed by strict barrier protection nursing and meticulous wound care without debridement using nanocrystalline silver dressings (Acticoat^®^). Antibiotics were not used prophylactically unless there was a clinical indication. Expert opinion was sought from the ophthalmologists on admission and instructions were carried out as per the ophthalmologist care plan. Their management plan entailed the use of a topical steroid (Maxitrol^®^), lubricants and glass rodding to prevent adhesions. Obstetricians and physicians were consulted when indicated.

### Data analysis

Data were analysed using Stata 13.0 SE (StataCorp. 2013. Stata Statistical Software: Release 13. College Station, TX: StataCorp LP). Descriptive statistics included means (standard deviations) for continuous variables and frequencies (*n* [%]) for categorical variables. Relationships between continuous predictors and dichotomous outcomes were assessed using the standard *t*-test or non-parametric Wilcoxon rank-sum test if the normality assumptions were violated. Similarly, analysis of variance (ANOVA, or non-parametric equivalent, namely the Kruskal–Wallis equality-of-populations rank test) was employed to compare the means of continuous predictors across the three-drug reaction groups. Correlation between continuous variables was assessed using the Spearman rank correlation coefficient. Differences in frequencies of categorical explanatory variables by drug reaction category were assessed using the Pearson chi-square (χ^2^) test or Fisher’s exact test if an expected cell count contained fewer than five observations. A *p*-value of < 0.05 was deemed statistically significant.

## Ethical consideration

Ethical approval was obtained from the University of KwaZulu-Natal Biomedical Research Ethics Committee (reference number BE417/14).

## Results

Of the 36 patients in the study, 32 (88.9%) were women. Eleven had SJS, 13 had SJS/TEN overlap and 12 had TEN. The number of days of hospitalisation increased exponentially along the spectrum of the disease (Table 2).

**TABLE 2:** Demographics of 36 HIV-infected patients presenting with Stevens–Johnson syndrome–toxic epidermal necrolysis.

Variable	Total (*n* = 36)				SJS (*n* = 11)				SJS/TEN (*n* = 13)				TEN (*n* = 12)			
	*n*	%	s.d.	Mean	*n*	%	s.d.	Mean	*n*	%	s.d.	Mean	*n*	%	s.d.	Mean
**Age (years)**																
< 40 years	30	83.3	-	-	10	27.8	-	-	10	27.8	-	-	10	27.8	-	-
> 40 years	6	16.7	-	-	1	2.8	-	-	3	8.3	-	-	2	5.6	-	-
**Mean (s.d.) age**																
Male	-	-	7.6	34.3	-	-	-	34.3	-	-	-	-	-	-	-	-
Female	-	-	10.4	32.4	-	-	-	27.1	-	-	-	34.8	-	-	-	33
**Sex**																
Male	4	11.1	-	-	4	11.1	-	-	0	-	-	-	0	-	-	-
Female	32	88.9	-	-	7	19.4	-	-	13	36.1	-	-	12	33.3	-	-
Length of hospital stay (Mean s.d.) no. days	-	-	11.2	15.1	-	-	2.4	5.8	-	-	6.9	13.0	-	-	11.3	25.8
**Pregnant patients (*n* = 32)**																
Y	16	50	-	-	5	31.3	-	-	5	31.3	-	-	6	37.5	-	-
N	16	50	-	-	2	12.5	-	-	8	50	-	-	6	37.5	-	-

SJS, Stevens–Johnson syndrome; TEN, Toxic epidermal necrolysis; SJS, TEN overlap; Y, yes; N, no; Regimen 1: NVP, nevirapine; 3TC, lamivudine; D4T, stavudine; TMP/SMX, trimethoprim/sulfamethoxazole; TB, tuberculosis; N/A, not applicable.

Sixteen (50.0%) women in the study were pregnant. The mean (s.d.) CD4 cell count in the pregnant women was 267.2 (60.6) cells/mm^3^. A significant number of pregnant women (93.8%) developed SJS/TEN secondary to nevirapine, while one was due to isoniazid prophylaxis. The pregnant women presented at a mean (s.d.) of 29.13 (3.76) weeks gestation. Five (31.25%) of the women delivered prematurely as a result of foetal distress, and all five of these patients had TEN. One of the deliveries was a stillbirth at 34 weeks’ gestation. The other four infants were healthy and showed no signs of any drug reaction.

CD4 cell counts were available for all the patients but one (Table 3). There was no association between the CD4 cell count and SCORTEN score (Spearman rho correlation test of 0.039). There was no significant difference in the mean CD4 cell count by drug type with an ANOVA *p*-value of 0.594 (data not shown).

**TABLE 3:** CD4, SCORTEN, drugs implicated, co-morbidities and mortality of 36 HIV-infected patients presenting with Stevens–Johnson syndrome–toxic epidermal necrolysis.

Variable	Total (*n* = 36)				SJS (*n* = 11)				SJS/TEN (*n* = 13)				TEN (*n* = 12)			
	*n*	%	Mean	s.d.	*n*	%	Mean	s.d.	*n*	%	Mean	s.d.	*n*	%	Mean	s.d.
**CD4 count (cells/mm^3^)**	-	-	236.8	146.8	-	-	216.3	109.2	-	-	270.5	194.8	-	-	217.4	115.5
**SCORTEN score**																
0-1	11	30.5	-	-	11	30.5	-	-	0	-	-	-	0	-	-	-
2	12	33.3	-	-	0	-	-	-	8	22.2	-	-	4	11.1	-	-
3	9	25.0	-	-	0	-	-	-	4	11.1	-	-	5	13.9	-	-
4	3	8.3	-	-	0	-	-	-	1	2.8	-	-	2	5.6	-	-
5	1	2.8	-	-	0	-	-	-	0	-	-	-	1	2.8	-	-
**Drugs used**																
NVP alone	15	41.7	-	-	7	19.4	-	-	3	8.3	-	-	5	13.9	-	-
NVP, D4T, 3TC/TMP/SMX	6	16.7	-	-	1	2.8	-	-	2	5.6	-	-	3	8.3	-	-
NVP, D4T, 3TC/Rifafour	2	5.6	-	-	1	2.8	-	-	0	-	-	-	1	2.8	-	-
NVP, D4T, 3TC/INH	1	2.8	-	-	0	-	-	-	1	2.8	-	-	0	-	-	-
NVP, D4T, 3TC/INH/TMP/SMX	1	2.8	-	-	0	-	-	-	1	2.8	-	-	0	-	-	-
TMP/SMX	2	5.6	-	-	1	2.8	-	-	1	2.8	-	-	0	-	-	-
TMP/SMX + phenytoin	1	2.8	-	-	0	-	-	-	0	-	-	-	1	2.8	-	-
TMP/SMX + Rifafour	3	8.3	-	-	0	-	-	-	1	2.8	-	-	2	5.6	-	-
Rifafour	3	8.3	-	-	1	2.8	-	-	2	5.6	-	-	0	-	-	-
Phenytoin	1	2.8	-	-	0	-	-	-	0	-	-	-	1	2.8	-	-
Fluconazole	1	2.8	-	-	0	-	-	-	1	2.8	-	-	0	-	-	-
**Comorbidity**																
TB	8	22.2	-	-	2	5.6	-	-	3	8.3	-	-	3	8.3	-	-
Hypertension	2	5.6	-	-	0	-	-	-	1	2.8	-	-	1	2.8	-	-
Epilepsy	2	5.6	-	-	0	-	-	-	1	2.8	-	-	1	2.8	-	-
**Mortality**	1	2.8	-	-	0	-	-	-	0	-	-	1	2.8	-	-

The mean (s.d.) CD4 cell count and confidence interval noted in patients with complications were 236.8 (186.4–287.2) cells/mm^3^. There was no statistical difference across all complications. Thus, the complications seen in the patients with SJS/TEN were not influenced by the CD4 cell counts and hence the level of immunosuppression in the sample.

The average SCORTEN scores for SJS, SJS-TEN overlap and TEN were 1, 2 and 3, respectively. There was a significant difference in the median SCORTEN score by drug reaction type *p* < 0.001. Thus, the more severe the drug reaction, the higher the SCORTEN score observed.

Comorbidities included hypertension, tuberculosis and epilepsy (Table 3). Ten (27.8%) of the patients in the study reacted to the anti-tuberculosis therapy, two due to isoniazid prophylaxis and the other eight to Rifafour^®^. Of these eight patients, only three were microbiologically confirmed cases of tuberculosis. The three patients were rechallenged with anti-tuberculosis therapy without further incident.

The most common drugs implicated were nevirapine (69.4%), anti-tuberculosis medication (16.7%) and trimethoprim/sulfamethoxazole (8.3%). Other drugs included phenytoin (2.8%) and fluconazole (2.8%) (Table 3).

Thirty-two of the study patients had associated complications such as anaemia, drug-induced hepatitis, ocular involvement, renal impairment, deep vein thrombosis, respiratory distress, lichen planus, Leucopenia, gastritis and hypernatremia. The number of complications noted increased along the continuum of the drug reactions with seven complications noted in SJS patients, 19 in SJS-TEN overlap syndrome and 27 noted in the patients with TEN (Table 4). Ten patients (28.7%) were noted to have infections (Table 5).

**TABLE 4:** Complications correlated to the various drug reactions.

Complications	SJS		SJS-TEN overlap		TEN		Total	
	*n*	%	*n*	%	*n*	%	*n*	%
Infection	1	2.8	4	11.1	5	13.9	10	27.8
Anaemia	3	8.3	5	13.9	8	22.2	16	44.4
Drug-induced hepatitis	2	5.6	4	11.1	3	8.3	9	25
Deep vein thrombosis	0	-	0	-	2	5.6	2	5.6
Lichen planus	0	-	1	2.8	0	-	1	2.8
Ocular	1	2.8	4	11.1	3	8.3	8	22.2
Renal impairment	0	-	1	2.8	2	5.6	3	8.3
Hypernatraemia	0	-	0	-	1	2.8	1	2.8
Leucopenia	0	-	0	-	1	2.8	1	2.8
Acute respiratory distress syndrome	0	-	0	-	1	2.8	1	2.8
Gastritis and esophageal ulceration	0	-	0	-	1	2.8	1	2.8

**Total**	**7**	-	**19**	-	**27**		**53**	-

SJS, Stevens–Johnson syndrome; TEN, Toxic epidermal necrolysis; SJS-TEN overlap.

**TABLE 5:** Profile of the 10 patients with infection, organisms isolated and SCORTEN score.

Drug reaction	CD4 (cells/mm^3^)	Infection	Organism	Comorbidity	SCORTEN score	Length of hospital stay (days)
TEN	268	Skin infection	*Pseudomonas aeruginosa* (pus swab)	TB	2	20
TEN	302	Pneumonia	MRSA (ET tube)	-	4	35
SJS/TEN	179	Genital ulcers	Herpes simplex	28 weeks pregnant	2	15
SJS/TEN	226	UTI	*E. coli* (urine sample)	29 weeks pregnant	2	9
TEN	247	Skin infection	*Pseudomonas aeruginosa* (pus swab)	34 weeks pregnant	3	20
SJS	0	PV discharge Genital warts	Candidiasis HPV	30 weeks pregnant	1	4
TEN	437	Skin infection	*Staphylococcus aureus* (pus swab)	-	2	24
SJS/TEN	10	Lower respiratory tract infection	*Pseudomonas aeruginosa* (blood cultures)	-	3	18
TEN	185	Pneumonia	*Staphylococcus aureus* (blood cultures)	32 weeks pregnant	4	Died on day 4 post admission
SJS-TEN	237	Bartholin abscess	*E. coli* (aspirate of abscess)	29 weeks	2	18

SJS, Stevens–Johnsons Syndrome; TEN, Toxic Epidermal Necrolysis; SJS-TEN overlap; MRSA, methicillin resistant staphylococcus aureus; ET tube, endotracheal tube; UTI, urinary tract infection; *E. coli, Escherichia coli*; PV discharge, per vaginal discharge; HPV, Human papilloma virus.

There was one death in our study representing a case fatality rate of 2.8%. The patient was pregnant (34 weeks gestation)and was admitted with TEN secondary to nevirapine. She had a CD4 count of 185 cells/mm^3^ and a SCORTEN score of 4 which predicted a poor outcome (expected mortality rate of 58.3%). She experienced preterm labour 2 days after admission and gave birth to a stillborn male. She died 4 days after admission. She presented with poor prognostic factors: SCORTEN score of 4 (tachycardia, BSA involvement of 40% acidosis and elevated urea, as well as hypernatremia and *Staphylococcus aureus* septicaemia).

## Discussion

This retrospective cohort study has shown that the use of systemic corticosteroids together with IVIGs for the treatment of SJS/TENS in HIV-infected patients resulted in a 97.2% survival rate compared to the previous report, which has a mortality rate of 30.0%.^4^ We observed that CD4 cell counts and SCORTEN score did not impact the mortality rate in HIV-infected patients.

The use of systemic corticosteroids in SJS/TEN has been an issue of debate for many years, and its use in HIV-infected patients remains highly controversial as it is thought to cause further immunosuppression.^22^ There have been case reports of HIV-infected patients being treated with systemic steroids and IVIG with a favourable outcome.^22,23,24,25^ Systemic corticosteroids in SJS/TEN is beneficial if used in the acute stage, that is, within 3–4 days of the disease onset and for short time periods, for less than a week.^12,26^ Hirahara et al. measured pro-inflammatory cytokines IFN ˠ, TNF α, interleukin 6 and 10 before and after high-dose methylprednisone therapy.^27^ This study showed that there was a significant decrease in the cytokines post therapy which could contribute to the survival of these patients.^27^ There are a number of other studies that support the use of systemic corticosteroids in SJS/TEN.^18,28,29,30,31^ High-dose corticosteroids early in the course of the disease decrease epidermal damage, shorten the recovery period and prevent permanent sequelae.^32^ Corticosteroids have been noted to decrease the intensity of the reaction, control the extension of necrolysis, decrease fever and discomfort and prevent damage to internal organs.^1^ This is supported by our findings. Kardaun et al. noted that dexamethasone therapy in SJS/TEN is more efficacious than long term lower dose therapy with a diminished risk of infection and delayed wound healing.^12^

Those opposing the use of systemic steroids argue that systemic steroids impair the immune system and increase the risk of infections.^33,34^ However, Aberdien et al. noted that systemic steroids are beneficial in the acute stage of infection in HIV-infected patients with *Pneumocystis jirovecii* pneumonia, acute bacterial meningitis, tuberculous pericarditis and meningitis and in patients with septic shock.^35^ A study by Mayosi et al. investigating the role of oral prednisone in HIV-associated tuberculous pericarditis demonstrated an insignificant effect on mortality, cardiac tamponade requiring pericardiocentesis and constrictive pericarditis. However, the incidence of constrictive pericarditis was significantly reduced by adjunctive corticosteroids (4.4% vs. 7.8%; *p* = 0.009), but this also resulted in an increase in HIV-associated malignancy (Kaposi sarcoma).^36^

The rationale for the use of systemic steroids in SJS/TEN is mainly due to anti-inflammatory and anti-apoptotic effects.^12,37^ There were no adverse effects noted at 3-month follow-up of our patients. We thus contend that this dose and the duration of systemic corticosteroids is unlikely to cause deleterious side effects.

Studies that have opposed the use of systemic corticosteroids are of the view that systemic corticosteroids are associated with a high rate of sepsis, poor wound healing, prolonged hospital stay and a higher mortality rate.^11,33,34,38^ Rasmussen reported a retrospective analysis of 32 immunocompetent children with SJS, 17 of whom were treated with systemic steroids for an unknown duration and of those treated with systemic steroids, 9 developed complications ranging from severe infections, seizures, gastrointestinal bleeds, pulmonary effusion and cushingoid facies.^33^ Based on the side effects noted by Rasmussen, a prolonged use of systemic steroids can be inferred. In a study of 30 patients by Helebian et al. all of whom were treated in a burns unit, 15 patients received supportive care alone while the other 15 received dexamethasone at various doses together with supportive care. There was a 66% mortality rate in the dexamethasone group as compared to the 33% mortality rate in the supportive group alone.^34^ Helebian et al. did not stipulate the dose of corticosteroid or the duration of use. This may account for the high mortality noted. Their skin care regimen also changed after the high mortality was noted in the group treated with systemic steroids. The positive outcome may not be a result of simply omitting systemic steroids but may also be due to a more intensive skin care regimen.

Studies have shown that IVIG arrests disease progression and reduces the time of skin healing.^14^ Two case reports by Tan et al. noted that IVIG administered to HIV-infected patients with TEN lowered morbidity and shortened the duration of hospital admission.^22^ In contrast, Brown et al. showed that there was no therapeutic benefit in using IVIG.^39^ This study showed a higher mortality rate of patients receiving IVIG than in controls and concluded that there is no role of IVIG in the setting of TEN and this should not be used outside of trials.^39^

Recent studies have shown that a combination of corticosteroids and IVIG arrested disease progression, and decreased hospitalisation and mortality in patients with SJS/TEN more than the use of corticosteroids as monotherapy.^2^ Jagadeesan et al. noted that combining systemic steroids and IVIG may have a synergistic action targeting the different pathways of apoptosis active in SJS/TEN.^37^ Thus, combination therapy was noted to arrest disease progression and there was a faster onset of re-epithelialisation with no adverse side effects.^37^ A systematic review showed that patients who received systemic steroids and IVIG had a favourable clinical outcome compared to patients who received supportive care alone.^40^

Local studies oppose the use of systemic steroids and immunoglobulins in patients with SJS/TEN. Kannenberg et al. conducted a study in which 78.9% of the patients were HIV-infected. All patients in the study received extensive supportive care. There was a mortality rate of 29.8% in the HIV-infected patients and 6.0% in the non-infected patients. The prognostic indicators noted were HIV -tuberculosis co-infection, sepsis and BSA > 40.0%.^9^ Knight et al. implemented similar treatment strategies of extensive supportive care in their study. Seventy-eight per cent of their cohort was HIV-infected. The study concluded that the extent of BSA involvement increases the risk of bacterial skin infections and that tuberculosis co-infection and bacterial skin infections increase the mortality rate. This study had a mortality rate of 9%.^41^

Many view TEN and major burns as similar entities based on BSA involvement, and hence the general principles of management should be the same.^13^ However, burns differ in pathophysiology, presentation, long term sequelae; hence, target therapy to counteract the process of apoptosis such as the use of systemic corticosteroids and IVIG is indicated for SJS/TEN. The admission of patients to specialised dermatology centres has shown good clinical outcomes, and this is illustrated in our cohort.^18^

None of the patients in this cohort had wound debridement, and this is supported by a number of studies.^1,19^ Blisters act as a natural biological dressing which favours re-epithelialisation.^42^ Fluid management also differs between SJS/TEN and burns patients in that SJS/TEN patients require only two-thirds to three-quarters of the fluid requirements of burns patients.^43^

Infection is the leading cause of death in patients with SJS/TEN and maybe as high as 40% in tertiary centres.^41^ Knight et al. noted that epidermal detachment > 30% in SJS/TEN has an increased risk of bacterial infections and mortalities.^41^ One of our patients with TEN had tuberculosis and *Pseudomonas* co-infection with a positive outcome. De Prost et al. noted that BSA involvement was the main predictor of infection.^44^ This group identified *Staphylococcus aureus, Pseudomonas* and *Enterobacteriaceae* as the most commonly implicated pathogens leading to mortality.^44^ These were similar pathogens noted in our patients. Complications noted in our patients who died are unlikely to have been due to the administration of systemic corticosteroids and IVIG. We receive patients that may have been at more than one health care facility prior to being transferred to our unit. Nosocomial infections may have been acquired at the referral centres prior to systemic corticosteroid use. Infections noted in the pregnant women included herpes simplex, vaginal discharge and vaginal warts. Urinary tract infections are common in pregnancy and may be independent to the use of systemic corticosteroids. Cutaneous *Pseudomonas* and *Staphylococcus aureus* infection may have been owing to the immunocompromised pregnancy state and HIV infection and may or may not be dependent on the use of systemic corticosteroids. Anaemia in one patient may be due to HIV disease, blood loss from the wounds, pregnancy and renal impairment. Drug-induced hepatitis was most likely due to nevirapine and trimethoprim/sulfamethoxazole use as the liver dysfunction returned to normal once the drugs were stopped. Infectious hepatitis was excluded in the study population.

Ocular lesions are the most common and devastating complications in SJS/TEN (20% – 79% of patients).^45^ Araki et al. noted that steroid pulse therapy with methylprednisone at the onset of SJS/TEN is of great therapeutic importance in preventing ocular complications.^46^ Kim et al. noted that early treatment with systemic steroids and IVIG improved ocular outcomes.^47^ The aetiology of deep vein thrombosis is multifactorial, and in one patient it may have been due to prolonged hospital stay, HIV infection, low CD4 cell count, pregnancy or IVIG used.^48,49,50^

In our study, the high female-to-male ratio may be due to the use of nevirapine which is implicated as the cause of SJS/TEN. It was prescribed in pregnant women (who accounted for 44.4% of the study sample) as part of the HIV treatment guidelines at the time of the study.

The three drugs most implicated included nevirapine, trimethoprim/sulfamethoxazole and anti-tuberculosis medication – mainly isoniazid and rifampicin. Risk factors for nevirapine-induced SJS/TEN include female gender, baseline CD4 counts > 250 cells/mm^3^ in women and > 400 cells/mm^3^ in men, history of drug allergy, low body weight, high nevirapine serum levels and certain human lymphocyte antigen types.^5,51,52^ Pregnant woman in the study had a mean CD4 count of 267.2 (s.d. 60.6) cells/mm^3^, a risk factor in keeping with nevirapine-induced SJS/TEN as noted in the literature.

Pregnancy was not a risk factor for developing a drug reaction in our patient profile. This is contrary to what has been suggested in the literature.^5,24^ Pregnancy is associated with immune dysregulation, which may predispose a woman to SJS/TEN.^24^ There was no statistically significant difference between CD4 cell count and SCORTEN score between the pregnant and the non-pregnant women. Thus, the severity of immunosuppression and severity of the drug reaction and predicted mortality was not influenced by pregnancy.

SCORTEN score directly correlated to the severity of the drug reaction. There was no significant correlation between the CD4 cell count and SCORTEN score, the severity of the drug reaction and the various complications. This could be explained by the fact that CD8 cytotoxic T cells are the main cells resulting in keratinocyte apoptosis.^13,53^ Yang et al. noted that HIV infection depleted the CD4+ regulatory cells (CD4+CD25+), and this resulted in the unregulated activity of the CD8 cytotoxic T cells which resulted in TEN.^54^

Marks et al. noted that drug reactions to anti-tuberculosis therapy occurred in 13.0% of the non-HIV-infected patients and 27.0% of the HIV-infected patients.^55^ Patients with SJS/TEN are often co-infected with tuberculosis, which further increases the mortality rate.^9^ Ten of the patients in our study group (27.8%) reacted to anti-tuberculosis treatment, and this was very similar to Marks et al.’s findings. Co-infection with TB was not associated with mortality in our cohort.

The limitations of this study are the retrospective study design and the small sample of patients. Future recommendations would be for larger randomised controlled studies to confirm the role of short course systemic corticosteroids in the management of HIV-associated SJS/TENS.

## Conclusion

This retrospective study has shown that an intensive skin care regimen in combination with systemic corticosteroids and/or IVIG in HIV-infected SJS and TEN patients, respectively, treated in a tertiary dermatology ward resulted in a 97.2% survival rate. Short course (3 days) systemic steroids were not associated with significant mortality in this HIV-infected cohort. A randomised controlled study is needed to confirm the results of this study.
